# Efficacy of Coblation Annuloplasty in Discogenic Low Back Pain: A Prospective Observational Study

**DOI:** 10.1097/MD.0000000000000846

**Published:** 2015-05-21

**Authors:** Liangliang He, Xiangyu Hu, Yuanzhang Tang, Xiuhua Li, Shuyue Zheng, Jiaxiang Ni

**Affiliations:** From the Department of Pain Management, Xuanwu Hospital, Capital Medical University, No. 45 Changchun Street, Xicheng Zone, Beijing, China.

## Abstract

In degenerative disc, the innervated outer annulus is confirmed to the major origin resulted in discogenic pain. To alleviate the discogenic pain, annuloplasty with electrothermal technology was proved to be effective, which mainly involves the thermal heating of the annulus to denature collagen fibers and denervate posterior annular nerve fibers. However, little is known that efficacy of annuloplasty with coblation technology in treating discogenic pain through directly interrupting nerves in outer annulus.

The purpose of this study was to evaluate the clinical outcomes of coblation annuloplasty for the treatment of discogenic low back pain.

In a clinical prospective observational study, 17 consecutive patients with discogenic low back pain underwent coblation annuloplasty under local anesthesia. Pain visual analogue scale (VAS) scores, patient responses stating significant (≥50%) pain relief, and modified MacNab criteria were adopted to evaluate the pain intensity, degree of pain relief, and functional status after 6 months of follow-up.

The preoperative pain VAS score was 6.5 ± 0.8(95% confidence interval [CI] 6.1–6.9) and the pain VAS score decreased to 2.9 ± 1.6 (95% CI 2.1–3.8), 2.9 ± 1.7 (95% CI 2.1–3.8), 3.2 ± 1.6 (95% CI 2.4–4.1), 3.2 ± 1.7 (95% CI 2.4–4.2) at 1 week and 1, 3 and 6 month postoperatively, respectively. 12 (70.6%), 11 (64.7%), 10 (58.8%) and 10 (58.8%) of patients reported significant pain relief at 1 week and 1, 3 and 6 months postoperatively. At 1, 3, and 6 months postoperatively, the numbers of patients with “excellent” or “good” ratings were 13 (76.5%), 11 (64.7%), and 10 (58.8%) according to the modified MacNab criteria. No serious complications were observed.

The finds show that coblation annuloplasty is an effective, safe, and less uncomfortable procedure in managing discogenic low back pain.

## INTRODUCTION

In a normal lumbar disc, nerve fibers mainly innervate the periphery of the outer annulus^1,2^; and in a degenerative disc, the nerve fibers maybe penetrate into the nucleus with aging and injury.^3^ If the innervations were irritated by biochemical or biomechanical stimulation,^4–7^ the disc itself can generate pain, which was defined as disocgenic pain. Although discogenic pain may be generated from irritated nerves in annulus or nucleus, the outer annulus was confirmed as the major origin through discography.^8^

To alleviate discogenic pain, annuloplasty was proved to be effective.^9–13^ The concept of annuloplasty originated from the technique of intradiscal electrothermal therapy (IDET) reported by Saal and Saal in the 2000s, which mainly involves the thermal heating of the annulus to denature collagen fibers and denervate posterior annular nerve fibers.^14^ And then, in the 2012's review of “effectiveness of thermal annular procedures in treating discogenic low back pain”, the results showed fair evidence for IDET in treating discogenic low back pain.^15^ However, the technical difficulties and time involved in threading a curved wire around the annulus were the major drawbacks, which limited the application of this procedure.

Present, coblation nucleoplasty as a minimally invasive procedure using radiofrequency energy to ablate nucleus was performed to alleviate discogenic pain through lowering intradiscal pressure and interrupting ingrown nerve endings along pathologic annular tears.^16–18^ However, the benefit in treating discogenic pain from nucleoplasty was limited, due to the scope of interruption of nerves maybe hardly reach to the outer annulus. Whereas, rare literatures reported that coblation annuloplasty was performed to manage discogenic pain. Based on its advantages of easer in technical operation, more security in therapeutic temperature and directly interrupting nerves in outer annulus, we hypothesized that coblation annuloplasty can relieve discogenic low back pain secondary to contained disc herniation.

## METHODS

### Patients

After obtaining the approval of the institution's Ethics Examining Committee of Human Research (Xuanwu Hospital, Capital Medical University, Beijing, China) and written informed patient consent, 17 patients mainly complained of discogenic low back pain secondary to contained disc herniation scheduled to receive coblation annuloplasty between October 2013 and June 2014 at Xuanwu Hospital.

Inclusion criteria for the coblation annuloplasty were as follows: unilateral discogenic low back pain without radicular pain and no neurological deficits, such as sensory or motor deficits or loss of reflexes; the pain VAS ≥ 4; the duration of pain ≥ 3 months; short-term or no treatment response to conservative management, including medication, physical therapy, and fluoroscopically directed injection therapies (lumbar medial branch block and lumbar epidural injection); contained disc herniation ≤ 6 mm and not compromising ≥1/3 of the central spinal canal according to magnetic resonance imaging (MRI)—the MRI scans must show an abnormal nucleogram with annular disruption in L4/5, L5/S1, or both and disc height ≥ 50%; a positive one-level provocation discography and fulfillment of criteria for a positive disc with provocation of concordant pain with an intensity of at least 7 of 10.

Patients affected by coagulopathy, uncontrolled psychological disorders, disc herniation with sequestration, infection, spinal instability, spinal fractures, tumor, advanced spondylosis resulting in osseous foraminal stenosis, or disc space collapse, as well as those with previous spinal surgery on the same level, were excluded from the study.

### Procedure

The procedure was performed in an operating room using sterile technique. The patient was placed in lateral position on the operation table; a 10-cm cushion was placed under the lumbar. The patient received the vital sign monitoring and oxygen supply at 3 L per minute via nasal prong throughout the procedure. Before the procedure, an intravenous injection of etimicin (1.0 g) was administered as a prophylactic antibiotic. Patients received intravenous injection of fentanil (50 μg) and were able to respond if a nerve root was irritated by thermal or mechanical stimulation. All procedures were performed under local anesthesia.

**FIGURE 1:**
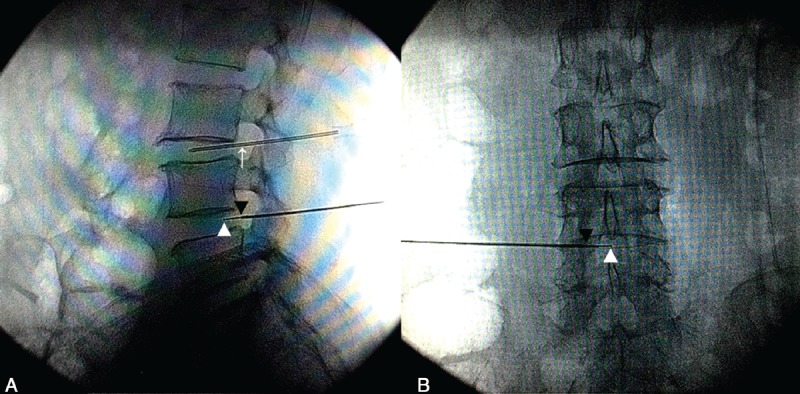
Intraoperative fluroscopic imaging. White short arrow indicates the tip of PERC-D wand in L4-5; black short arrow indicates the tip of cannula in L4-5; white long arrow indicates the assisted-orientation needle paralleled L3-4. (A) Lateral view. (B) Anterial-posterial View.

Under fluoroscopic guidance with anterior-posterior (AP) and lateral views and the assisted orientation of another metal needle, a 12-gauge, 15-cm cannula was advanced via a left or right posterolatral approach to the posterior margin of outer annulus. Cannula was advanced slowly and the advancement was stopped immediately when there was an increase of resistance signaling entry into the annulus. The resistance was check using loss of resistance with glass syringe. When cannula was advanced to only contact the posterior margin of outer annulus but not into annulus, the resistance was loss. Then, cannula was advanced into annulus slightly until the resistance occurred. A paresthesia may be elicited if the exiting nerve root was contacted. The cannula position was adjusted until the parethesia disappeared. During the process of cannula advancement, the cannula position should be check under the AP and lateral views, and the final position should be in the posterior outer layer of annulus, as shown in Figure 1.

The coblation wand (UNITEC, China America United Technology (Beijing) Co. Ltd, China) was inserted into the cannula and advanced slightly into the annulus. The position of the tip of wand was check again under AP and lateral views (Figure 1). The maximum treatment depth was 1 cm, which was marked with wand hub. Next, coagulation was tested with the radiofrequency controller set at 2’ for 1/2 to 1 second to check that there was no movement or paresthesia in the patient's lower limbs. A channel in annulus was created at a speed of 0.2 cm/sec in coblation mode with the radio-frequency controller set at 2’ of intensity, and then retracting the wand at a speed of 0.5 cm/sec in coagulation mode with the radio-frequency controller set at 2’ of intensity. Annuloplasty was accomplished by creating 6 channels in annulus at 12, 2, 4, 6, 8, and 10 o’clock position circumferentially. If resistance was encountered, wand movement should cease and its position should be fluoroscopically evaluated. If the resistance persisted, advancement should be stopped and shorter channels should be accepted at this position. After withdraw of the wand, 2 mL of 0.5% lidocaine was injected into the annuloplasty tract. All patients were subjected to bed rest in the supine position for 48 hours. After discharge from the hospital, patients were advised to avoid long-term bend and strenuous activities. All procedures were performed by one same surgeon who has >5 years experience in performing coblation technology in lumbar disc, which is helpful to avoid the technique resulting in clinical outcome bias.

### Therapeutic Efficacy Assessment

Clinical improvement of pain after coblation annuloplasty was qualified with a pain VAS score recorded preoperatively and at 1 week and 1, 3, and 6 months postoperatively. Significant pain relief (postoperative pain relief ≥50% compared with the preoperative state) was recorded at 1 week and 1, 3, and 6 months postoperatively. Patient's functional status was assessed with “excellent,” “good,” “fair,” and “poor” according to the modified MacNab criteria and recorded at 1, 3, and 6 months postoperatively. Complications, such as hemorrhages, paresthesia, and infection, were recorded.

### Statistical Analysis

The patients’ demographic and baseline clinical data were analyzed descriptively. Repeated measures analysis of variance (ANOVA: a parametric test) was used to compare the improvement in pain VAS scores between the preoperative and postoperative time points. The Wilcoxon signed-rank test was used to evaluate the amount of significant pain relief and the functional status of patients after 6 months of follow-up. A value of *P* < 0.05 was considered statistically significant in all analyses. Statistical analyses were performed using GraphPad Prism version 5.0 (GraphPad Software Inc, San Diego, CA).

## RESULTS

A total of 17 patients with discogenic low back pain undergone coblation annuloplasty, 11 males and 6 females. The mean pain VAS score was 6.5 ± 0.8 (range 5–8), mean age was 52 ± 7 year-old (range 36–63 year-old), and average duration of pain was 5 ± 3 years (range 0.5–12 years) (Table 1).

**TABLE 1:**
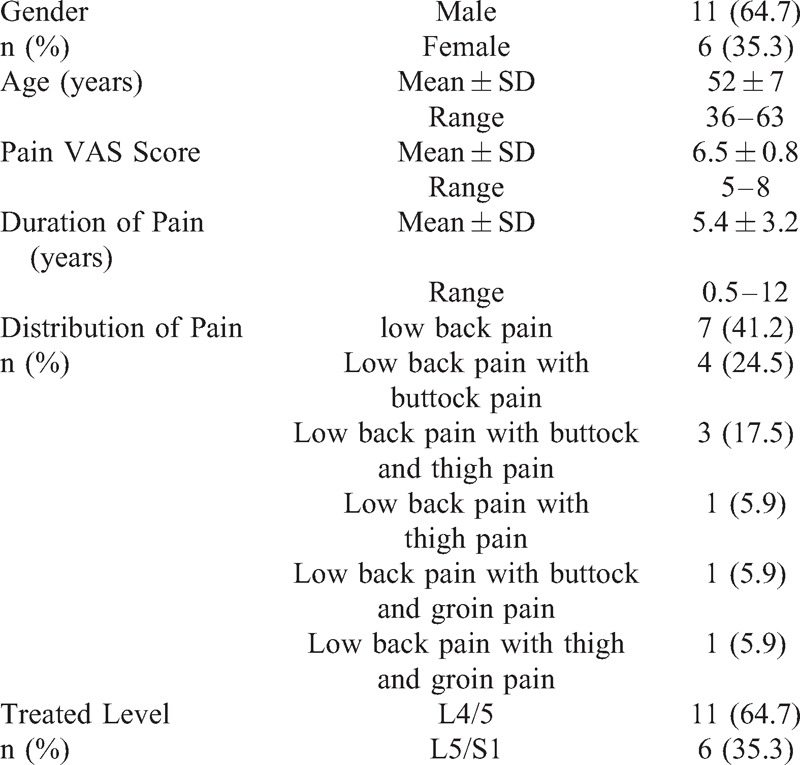
Demographic Characteristic

Before the operation, 7 patients complained of low back pain only, 4 patients complained of low back pain with buttock pain, 3 patients complained of low back pain with buttock and thigh pain, 1 patient complained of low back pain with thigh pain, 1 patient complained of low back pain with buttock and groin pain, 1 patient complained of low back pain with thigh and groin pain, the pain quality was described mainly as dull, sore, and swelling. Coblation annuloplasty was used to treat the L4/5 disc level in 11 cases (64.7%), and the L5/S1 disc level in 6 cases (35.3%) (Table 1).

**FIGURE 2:**
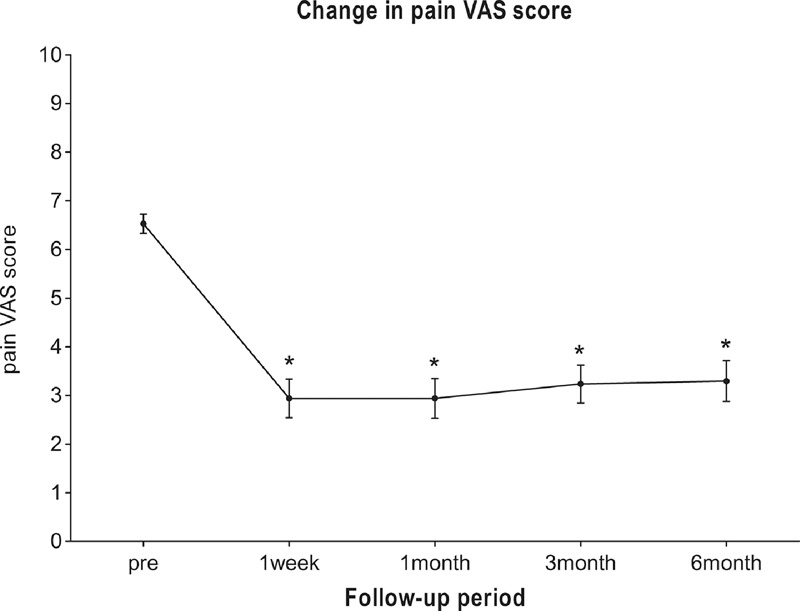
The pain VAS score preoperatively, and 1 week and 1, 3 and 6 months postoperatively. Values are shown as means (error bars: 95% CI for mean). ∗ indicates significant difference with pre-procedure value.

**FIGURE 3:**
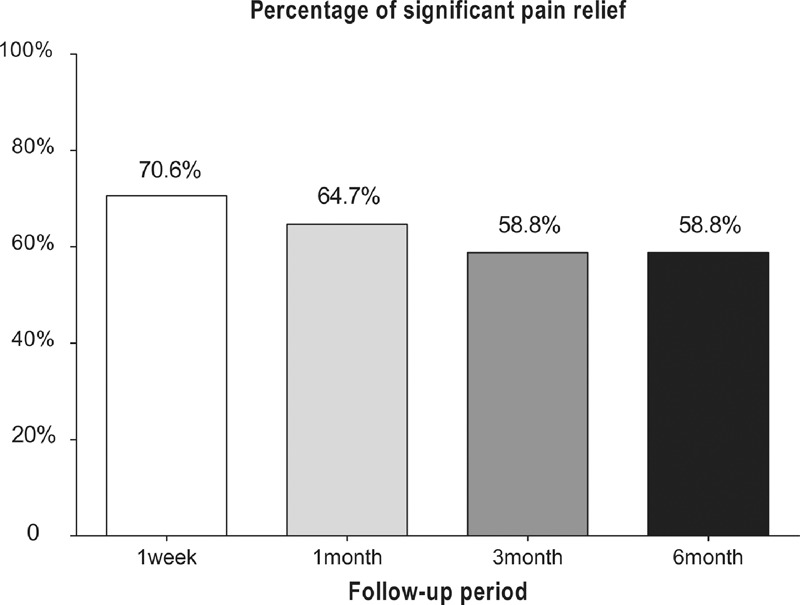
The proportion of patients reporting significant (≥50%) pain relief at 1 week and 1, 3 and 6 months postoperatively.

Compared with preoperation, the pain VAS score obviously decreased at 1 week and 1, 3, and 6 months postoperatively. The preoperative pain VAS score was 6.5 ± 0.8 (95% confidence interval [CI] 6.1–6.9) and the pain VAS score decreased to 2.9 ± 1.6 (95% CI 2.1–3.8), 2.9 ± 1.7 (95% CI 2.1–3.8), 3.2 ± 1.6 (95% CI 2.4–4.1), 3.2 ± 1.7 (95% CI 2.4–4.2) at 1 week and 1, 3, and 6 month postoperatively, respectively (Figure 2). Only 2 patients reported that the pain VAS score decreased to 0 during the period of post-procedure 6 months, but 12(70.6%), 11(64.7%), 10(58.8%) and 10(58.8%) of patients reported significant pain relief at 1 week and 1, 3 and 6 months postoperatively (Figure 3).

**FIGURE 4:**
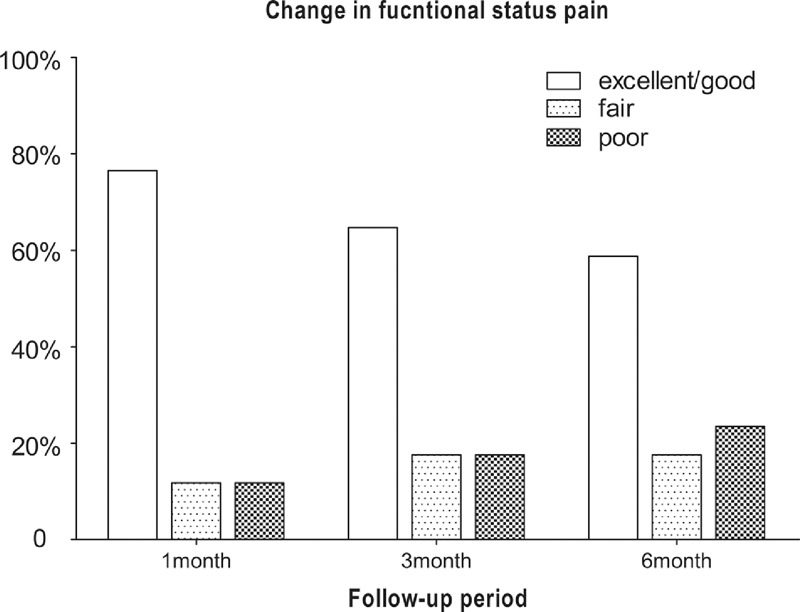
The proportion of patients who expressed “excellent” or “good”, “fair” and “poor” at 1, 3, and 6 months postoperatively.

According to the modified MacNab criteria, no difference was found in the proportion of patients with “excellent” or “good” ratings. At 1, 3, and 6 months postoperatively, the numbers of patients with “excellent” or “good” ratings were 13 (76.5%), 11 (64.7%) and 10 (58.8%); the numbers of patients with “fair” ratings were 2 (11.8%), 3 (17.6%) and 3 (17.6%); and the numbers of patients with “poor” ratings were 2 (11.8%), 3 (17.6%), and 4 (23.5%) (Figure 4).

After 6 months follow-ups, 7 patients didn’t experience significant pain relief: 2 patients with low back pain only preoperatively complained of low back pain, 1 patient with low back and buttock pain preoperatively complained of buttock pain only, 1 patient with low back pain, buttock and thigh pain preoperatively complained of buttock and thigh pain, 1 patient with low back, buttock and thigh pain preoperatively complained of thigh pain, 1 patient with low back and thigh pain preoperatively complained of no change in pain, 1 patient with low back, thigh and groin pain preoperatively complained of no relief in pain. Among above 7 patients, 5 patients needed to take anesthetics to relieve pain.

Among 17 patients, 1 patient with low back pain only preoperatively was observed to 12 months postoperatively and significant pain relief was reported; 2 patients were observed to 11 months postoperatively: significant pain relief in 1 patient with low back and buttock pain preoperatively and no pain relief in 1 patient with low back and thigh pain preoperatively; 2 patients were observed to 10 months postoperatively: significant pain relief and 30% to 40% of pain relief were reported respectively in 2 patients with low back pain only preoperatively; 1 patient with low back, thigh and groin pain preoperatively was observed to 9 months postoperatively and no pain relief was reported; 3 patients were observed to 8 months postoperatively: significant pain was found in 3 patients (1 patient with low back pain only,1 patient with low back and buttock pain, and 1 patient with low back, buttock and thigh pain preoperatively); 4 patients were observed to 7 months postoperatively: significant pain relief was reported in 2 patients (1 patient with low back pain only and 1 patient with low back and buttock pain preoperatively), 20–30% of pain relief was reported in 1 patient with low back, buttock and thigh pain preoperatively and 30–40% of pain relief was reported in 1 patient with low back and buttock pain preoperatively; 4 patients were observed to 6 months postoperatively: significant pain relief was found in 2 patients (1 patient with low back pain only and 1 patient with low back, buttock and groin pain preoperatively), and 30% to 40% of pain relief was reported in 1 patient with low back pain only preoperatively and 20% to 30% of pain relief was found in 1 patient with low back, buttock and thigh pain preoperatively.

Among 17 patients, 2 patients experienced ecchymoma and 5 patients reported soreness at the needle insertion site, but the symptoms completely disappeared within two weeks after operation. No hemorrhages, paresthesia, or infection were observed.

## DISCUSSION

In this study, >50% of patients expressed significant pain relief and “excellent” or “good” according to the modified MacNab criteria after 6 months follow-up. Remarkable reduction in discogenic low back pain and significant improvement in functional status were demonstrated after coblation annuloplasty.

Coblation technology is applied through a PERC-D wand, which has 1 mm diameter and is a bipolar instrument design. Through the wand, coblation technology can provide a therapeutic effect with two modes: coblation and coagulation. During coblation process, the tissue molecular bonds are breakup into various elementary molecular and low molecular weight gases with a highly focused plasma field, which is generated around the tip of wand in conductive medium with radiofrequency energy and creates small channel in tissues. During coagulation process, the adjacent tissue are thermally sealed, including shrinkage of collages. The advantage of ablation technology is replacing the thermally damaging vaporization with molecular dissociation at a low temperature ablative process (40–70°C).^16–18^

In 1999, the coblation technology was approved for use in spine by US Food and Drug Administration, and the coblation nucleoplasty was first performed in 2000. Later, the efficacy data of cobation nucleopalsty in treating contained disc herniation with associated symptoms were reported by a series of clinical studies.^19–23^ However, the efficacy data of nucleoplasty for lumbar discogenic pain was rare.^24,25^

In 2004, Singh et al^24^ published the clinical outcomes of coblation nucleoplasty in treating 47 patients with discogenic low back pain. The proportion of patients who reported ≥ 50% pain relief was 80%, 74% 63% and 53% at the 1, 3, 6 and 12 months follow-up, respectively. And the functional improvements were reported by 46% of patients for sitting ability, 41% for standing ability, and 49% for walking ability at postoperative 12 month. To 2013, Kumar et al^25^ again published the therapeutic role of nucleoplasty in the management of discogenic axial back pain on 30 patients. The clinical outcomes showed that significant pain relief (VAS decrease ≥ 30%) was 53.30% and 60%, significant reduction in the functional disability (Oswestry Disability Index (ODI) decrease ≥ 12.8%) was 93.30% and 80%, significant improvement in quality of life (ShortForm-36 [SF-36] increase ≥ 5%) was 96.7% and 83.30% at 6 and 12 month postoperatively.

However, more proponents and clinical evidence supported nucleoplasty for individual with lumbar radicular pain,^26,27^ because of decompression of nerve root through removing nucleus material, decreasing nucleus volume and lowering intradiscal pressure.^28,29^ In contrast, the benefit of coblation nucleoplasty in treating discogenic low back pain is uncertain, because the scope of interruption of nerves hardly reached to the outer annulus, which was considered as the major origin of discogenic pain.^8^

In this study, the therapeutic purpose of coblation annuloplasty is similar with IDET, which is to denature collagen fibers and denervate posterior annular nerve fibers.^14^ In previous studies of IDET, the pain VAS score obviously decreased from mean 6.1–7.4 preoperatively to 1.7–3 at 6 month postoperatively,^9–13^ which consistent with the results of our study, the pain VAS score significantly decreased from pre-operative 6.5 to 3.2 at 6 month postoperatively. In this study, the functional status was evaluated with the modified MacNab criteria, which was different from other study of IDET with ODI and SF-36 questionnaires,^11^ but similar clinical outcomes were proved. Although rare efficacy data of coblation annuloplasty was published, the initial clinical evidence supported that coblation annuloplasty appeared to be an alternative to IDET for treatment of discogenic low back pain was provided in our study.

During coblation annuloplasty process of this study, complete, part and no concordant pain was reproduced in 5, 7 and 5 patients, respectively. For the phenomenon of part concordant reproduced pain, the possible explanation is that the scope of interruption of nerves maybe only locate along access path of the tip of wand^17^, which is technical deficiency compared with IDET. However, interesting, among 5 patients with no concordant reproduced pain, 20% to 30% of pain relief was observed in 1 patient and over 50% of pain relief in 2 patients. The possible explanation is that lowering the tension in the outer annulus is benefit for decompression of nerves innervated the outer annulus.^28,29^ Additionally, the modification of biochemical state to inhibit inflammatory stimulation to nerves innervated the outer annulus maybe play one therapeutic role in treating discogenic pain.^30^

In our study, 2 patients experienced ecchymoma and 5 patients reported soreness at the needle insertion site, which has been reported as the most common side effect in coblation technology; however, the symptoms completely disappeared in two weeks after operation.^31^ No complications, such as hemorrhages, paresthesias, or infections, were observed in this study.

The limitation of this study is lack of control group, historic or placebo. Conducting a blind, randomized, placebo-control study may be prohibitively expensive and logistically difficult in a practice setting. The technique of coblation annuloplasty is maybe criticized in our study, because of the potential nerve injury due to the tip of wand placed on periphery of outer annulus, which is closed to traversing or exiting nerve root. However, different from other thermal annular therapies, coblation technology is a nonheat driven process.^16–18^ A thermal mapping study in the porcine model confirms a steep temperature drop off from the tip of wand. When the distance from the tip of wand is 1 mm and 5 mm, the temperature decreased to ≤20°C and 0°C, respectively.^32^ Additionally, the radius of the thermal zone of coagulation is approximately 1 mm when the wand is moved 0.5 cm/sec.^33^ Therefore, the security in temperature is the most important advantages in coblation technology. During the whole annuloplasty process, the patients were under light sedation and maintain protective reflexes in order to report immediately possibly nerve injury in our study.

Although the annulus as the target tissue to be treated with coblation technique in our study, the nucleus was irrelevantly be dealt in some cases during surgical process. But, we adopted the phrase “cobaltion annuluplasty” as being more accurately descriptive of the procedure.

## CONCLUSION

Coblation annuloplasty is an effective, safe, minimally invasive and less uncomfortable procedure for treatment of discogenic low back pain.
